# Zika Virus Infection and Antibody Neutralization in FcRn Expressing Placenta and Engineered Cell Lines

**DOI:** 10.3390/vaccines10122059

**Published:** 2022-11-30

**Authors:** Yanqun Xu, Yong He, Sanaz Momben-Abolfath, Devin Vertrees, Xiaohong Li, Malgorzata G. Norton, Evi Budo Struble

**Affiliations:** Laboratory of Plasma Derivatives, Division of Plasma Protein Therapeutics, Office of Tissues and Advanced Therapies, Center for Biologics Evaluation and Research, U.S. Food and Drug Administration, Silver Spring, MD 20993, USA

**Keywords:** Zika virus, flavivirus, anti-viral antibody responses, virus–host interactions, antibody-dependent enhancement (ADE), FcRn

## Abstract

As a developmental toxicant, Zika virus (ZIKV) attacks both the growing nervous system, causing congenital Zika syndrome, and the placenta, resulting in pathological changes and associated adverse fetal outcomes. There are no vaccines, antibodies, or other treatments for ZIKV, despite the potential for its re-emergence. Multiple studies have highlighted the risk of antibodies for enhancing ZIKV infection, including during pregnancy, but the mechanisms for such effects are not fully understood. We have focused on the ability of the neonatal Fc receptor (FcRn) to interact with ZIKV in the presence and absence of relevant antibodies. We found that ZIKV replication was higher in Marvin Darby Canine Kidney (MDCK) cells that overexpress FcRn compared to those that do not, and knocking down FcRn decreased ZIKV RNA production. In the placenta trophoblast BeWo cell line, ZIKV infection itself downregulated FcRn at the mRNA and protein levels. Addition of anti-ZIKV antibodies to MDCK/FcRn cells resulted in non-monotonous neutralization curves with neutralization attenuation and even enhancement of infection at higher concentrations. Non-monotonous neutralization was also seen in BeWo cells at intermediate antibody concentrations. Our studies highlight the underappreciated role FcRn plays in ZIKV infection and may have implications for anti-ZIKV prophylaxis and therapy in pregnant women.

## 1. Introduction

Zika virus (ZIKV) is a teratogen that adversely impacts the developing fetus via two pathways. It directly enters fetal compartment homing into neuronal progenitor cells and immature neurons [1,2], causing cell death and severe damage to the nervous system. This results in fetuses and infants with various anomalies which are collectively termed congenital Zika syndrome (CZS) [3]. ZIKV can also establish a productive [4,5] and long-lasting [6] infection in the placenta, targeting multiple cell types and resulting in placental pathology [7]. Dysfunction of the placenta can then occur, which can impact oxygen and nutrient exchange and result in additional adverse pregnancy outcomes, such as growth restriction and low birth weight of the newborn [8,9].

Vaccines and antibody treatments have been proposed and are being studied for ZIKV. For such modalities to be beneficial, they should disrupt both placental and fetal infections. However, we and others have reported that some antibodies may not effectively accomplish one or both tasks. Dengue cross-reactive antibodies may enhance placenta infection [10] or increase placental transfer of the infection and worsen fetal outcomes [11]. Even neutralizing antibodies may enhance viral entry into susceptible cells at certain concentrations [12]. Understanding the mechanisms underlying such processes is important as it could aid in designing safe and effective antibody-based prophylactic and therapeutic strategies, not only for ZIKV but also other viruses.

Here, we report our findings that the neonatal Fc receptor (FcRn) may both play a role in and be downregulated by ZIKV infection. This may have implications for the IgG antibody transfer and efficacy of the anti-ZIKV prophylaxis and therapy during ZIKV infection, given that FcRn plays an important role in placental transfer and half-life of IgG therapy.

## 2. Materials and Methods

### 2.1. Zika Virus and Cells

The Zika virus (Puerto Rican) strain PRVABC59 used in this study was isolated by the CDC from the serum of a ZIKV-infected patient who travelled to Puerto Rico in 2015. The infectious virus was grown in Vero E6 cells (ATCC) and purified as previously reported [12].

The BeWo (human choriocarcinoma cell line) clone b30 was a kind gift from Erik Rytting lab, University of Texas Medical Branch (UTMB). Marvin Darby Canine Kidney Cell line 2 (MDCK2) transfected with either human FcRn receptor (MDCK/FcRn) or the empty vector (MDCK/vector) were a kind gift from Richard Blumberg lab, Harvard Medical School. The cells were passaged (less than 30 passages) in DMEM, supplemented with 10% fetal bovine serum (FBS), and Antibiotic–Antimycotic mixture (AA, Thermo Fisher).

### 2.2. Antibodies

ZENV14 m (mAb14) and ZENV17 m (mAb17) were purchased from Alpha Diagnostic International (San Antonio, TX, USA) and used as before [12]. Briefly, mAb14 is a human IgG1 anti-ZIKV envelope protein, and mAb17 is a humanized IgG1 anti-flavivirus envelope protein.

The following antibodies were used for Western blots: mouse anti-FcRn sc-271745 (Santa Cruz Biotechnology Inc., Dallas, TX, USA), mouse anti-actin sc-56459 (Santa Cruz, CA, USA), and donkey-anti-mouse HRP A90-337P (Fortis Life Sciences, Waltham, MA, USA). Anti-FcRn antibody ABIN1774763 (Antibodies-online Inc., Limerick, PA, USA) was used for an antibody blockade of FcRn function.

### 2.3. Assessment of ZIKV Infectivity and Antibody Mediated Neutralization

Suspensions of MDCK/FcRn, MDCK/vector or BeWo cells in DMEM Glumax^®^ medium (Thermo Fisher), supplemented with 10% fetal bovine serum (FBS), non-essential amino acids (NEAA) and Antibiotic–Antimycotic mixture (AA, Thermo Fisher) were seeded in a flat-bottom 96-well plate and incubated at 37 °C overnight to reach 70–90% confluency. The following day, the media was replaced with ZIKV or ZIKV/antibody mixture to perform infectivity and neutralization assays, respectively. For infectivity assays, serially diluted ZIKV aliquots (3 times dilution series) were prepared using DMEM media supplemented with 2% FBS, starting at an approximate MOI of 3–6 for a total of 8 dilutions and added in quadruplicates to the cells; the last row was comprised of cells with no virus. For neutralization assays, mAb14 or mAb17 antibodies were serially diluted and mixed with an equal volume of the virus at MOI of one (1) in the same low (2%)-FBS DMEM as before, incubated at 37 °C for one hour, and then added in quadruplicates to the cells. The next day (neutralization assay) or 48 h post-infection (infectivity assay) the media was removed, and the cells were washed with PBS and assessed for the presence of infection. Briefly, a volume of 100 µL iScript sample preparation reagent (Bio-Rad) was added to each well, the plate was incubated at room temperature for 2–5 min, and the supernatant collected and used immediately for PCR analysis or stored at −80 °C until we were ready to test.

### 2.4. siRNA and Antibody Knockdown of FcRn Expression

The protocol was adapted from SantaCruz Biotechnology Inc. (Santa Cruz) and adjusted for 24-well tissue culture plate. FcRn and control siRNA, namely sc-45632 and sc-37007, respectively (Santa Cruz) were the same as reported [13]. Briefly, MDCK/FcRn or BeWo cells were seeded in 500 µL antibiotic-free normal growth medium supplemented with 5% FBS and incubated at 37 °C until the cells were 60–80% confluent. Then, the media was removed and cells were washed with PBS. For each transfection, 2 µL (i.e., 20 pmol) siRNA duplex in 40 µL transfection mixture was incubated at room temperature for 15–45 min, then mixed with 180 µL transfection medium (Santa Cruz) and overlaid onto washed cells. Tissue culture plates were subsequently incubated for 6 h in a 37 °C incubator, then 200 µL DMEM medium supplemented with 20% FBS was added. Transfection media mixture was replaced with 400 µL fresh normal growth (10% FBS) media the following day before cells were allowed to grow overnight and then infected with ZIKV at 1 or 3 × 10^5^ TCID_50_/well. 24 h after the addition of the virus, cells were collected to assess ZIKV infection and FcRn expression via quantitative RT PCR and FcRn protein expression with Western blot analysis. To prepare crude cell lysates, 300 µL iScript sample preparation reagent (Bio-Rad) was added in each well and incubated for 5 min then collected in fresh tubes and used for PCR or mixed with SDS loading buffer (NuPage LDS sample loading buffer and sample reducing agent, Invitrogen) for Western gel analysis, as described [12].

Alternatively, RNeasy mini kit (Qiagen) was used to extract RNA from BeWo cells following the manufacturer’s instruction, and the expression of FcRn was assessed via quantitative RT PCR.

For antibody blockade experiments, quadruplicates of MDCK/FcRn or BeWo cells in a 96-well plate were incubated with 10 µg/mL of either anti-FcRn antibody, an unrelated antibody or DMEM/2% FBS media for 1.5 h, then ZIKV at MOI 5-1.7 was added. Two days (48 h) post-infection cells were lysed with iScript sample preparation reagent as before and ZIKV RNA was measured using qRT PCR.

### 2.5. PCR

To assess the amount of ZIKV in crude cell lysates (infectivity or neutralization tests) or purified RNA (FcRn expression), one-step quantitative RT PCR was used (TaqMan^TM^ RNA-to-CT^TM^ 1-Step Kit from Applied Biosystems, www.thermofisher.com, accessed on 30 November 2022) using a Applied Biosystems QuantStudio 3 real-time PCR machine. ZIKV primers were chosen to allow for high sensitivity, as previously reported [12]. Human and canine GAPDH and human FcRn primer/probe sets were designed using an IDT primer ordering tool (idtdna.com, accessed 30 November 2022). All the primers used in this study are shown in Table 1.

Crude cell lysates and the purified RNA were diluted using nuclease free water at least 1:4 or 1:10, respectively, prior to performing PCR. Each 10 µL PCR reaction contained 4 µL template, 0.5 µM forward and reverse primers and 0.25 µM probe. All samples were run in duplicates or triplicates. For quantification of ZIKV, a standard curve was included or the ΔΔC_t_ method was used, as previously described [12]. For FcRn expression analysis, the ΔΔC_t_ method was used. For the ΔΔC_t_ method, the GAPDH housekeeping gene was chosen as the endogenous control.

### 2.6. Data Processing and Statistical Analysis

Absolute and relative ZIKV RNA expression levels from the C_t_ values were quantified using both a standard curve (as previously described [12]) and the ΔΔC_t_ method. Relative FcRn mRNA expression was assessed with the ΔΔC_t_ method. Human or canine GAPDH were the endogenous controls for the ΔΔC_t_ method for BeWo and MDCK/FcRn cells, respectively. ΔC_t_ values from MDCK/vector infection experiments were used as the baseline to calculate ΔΔC_t_ for MDCK/FcRn infection. ΔC_t_ from cells without infection or without siRNA were used to calculate ΔΔC_t_ for FcRn expression in infected and siRNA-treated cells, respectively. Differences in RNA expression levels were then calculated using the expression: Fold Change = 2^(−ΔΔCt)^.

Western blots were imaged using GBOX Mini (Syngene, syngene.com, accessed on 30 November 2022) imaging system and then analyzed with ImageJ (NIH open source, Version 1.53t). The area under the intensity peak curve for the FcRn band was calculated using software tools; this value was then normalized by dividing with the intensity of the corresponding actin band to allow for comparisons across experiments.

Both *t* tests or one-way ANOVA for column analyses and 2-way ANOVA for grouped analyses (GraphPad Prism 9) were used to assess the differences in expression levels, and *p* < 0.05 was considered significant.

## 3. Results

### 3.1. ZIKV Infectivity in MDCK Cells That Overexpress Human FcRn versus Those That Do Not

**Figure 1:**
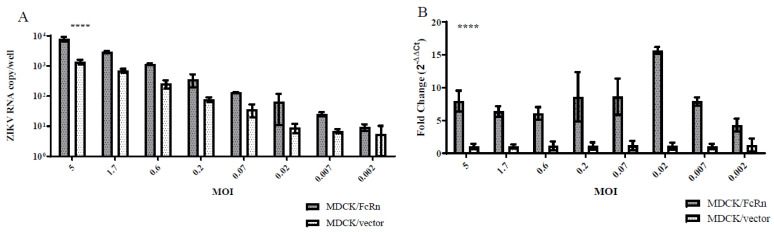
Marvin Darby Canine Kidney (MDCK) cells that overexpress human FcRn are more permissive of Zika virus (ZIKV) infection than those that do not. MDCK/FcRn (shaded bars) or MDCK/vector cells were infected with serial dilutions of ZIKV, and cellular ZIKV RNA expression at 48 h was quantified using either (**A**) a standard curve, or (**B**) an endogenous gene expression (canine GAPDH). The experiment was performed in quadruplicates for each of the 8 dilutions and data was analyzed using a two-tailed paired *t* test, comparing infection levels in the two cell lines at each MOI level, respectively, **** *p* < 0.0001.

**Figure 2:**
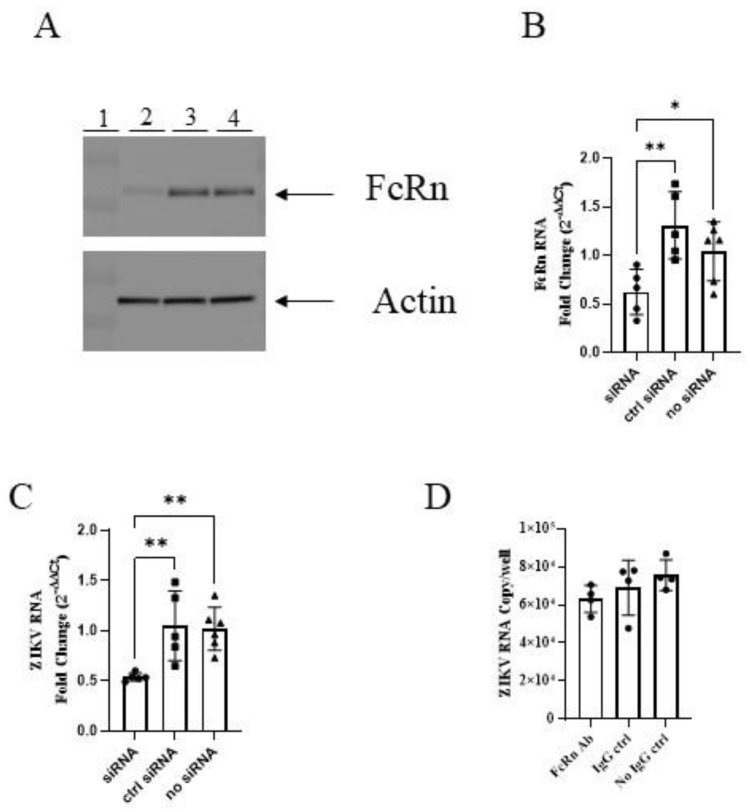
siRNA knockdown of FcRn in MDCK/FcRn cells reduces ZIKV infection. (**A**) Western blot demonstrating knock-down of FcRn receptor expression in MDCK/FcRn cells. Lane 1—molecular weight marker; 2—FcRn siRNA; 3—control siRNA; 4—no siRNA. (**B**) RT qPCR showing knock-down expression of FcRn at the mRNA level. (**C**) Less ZIKV mRNA is seen in cells with knock down expression of FcRn. One representative of two independent experiments is shown (**A**–**C**), with at least five biological repeats per each cell culture analyzed by PCR (**B**,**C**). (**D**) No statistical difference in ZIKV mRNA is seen in cells treated with anti-FcRn antibody before infection, although the levels are trending lower. One experiment with four biological repeats was performed. The data was analyzed with ordinary one-way ANOVA, * *p* < 0.05, ** *p* < 0.01.

We infected MDCK cells stably transfected with human FcRn (MDCK/FcRn) and those stably transfected with the empty vector (MDCK/vector) with ZIKV PRVABC59 strain at a wide range of MOIs and then measured the intracellular ZIKV RNA levels. The viral RNA levels in MDCK/FcRn were higher than in MDCK/vector cells at all the ZIKV dilutions tested. This was true, whether the standard curve (Figure 1A) or ΔΔC_t_ (Figure 1B) method was used to quantify ZIKV RNA. To further confirm that this difference was due to FcRn overexpression, we used siRNA technology to knock down the expression of this receptor and compare the infection levels in cells treated with FcRn siRNA, a control siRNA, and those that did not receive any siRNA treatment (Figure 2). As expected, adding siRNA which was specific to human FcRn resulted in a marked decrease in FcRn expression in the Western blot (Figure 2A) and a statistically significant reduction in FcRn mRNA expression (Figure 2B) compared to the controls. MDCK/FcRn cells with knocked-down expression of FcRn subsequently exhibited a significant reduction in ZIKV RNA levels (Figure 2C) compared to the controls. A smaller, not statistically significant trend of reduced infection was also seen when an anti-FcRn antibody was used (Figure 2D). We note that a statistically significant small increase was seen in the average RNA levels of FcRn in wells treated with control siRNA versus media-only controls (Figure 2B). Although we cannot rule it out, we are not aware of any mechanistic reasons that explain this non-specific effect. It is more likely that this finding is an artifact related to the variability of the assay, and it does not impact the interpretation of our data, especially since it was both of small magnitude and not observed at the protein level (Figure 2A).

**Figure 3:**
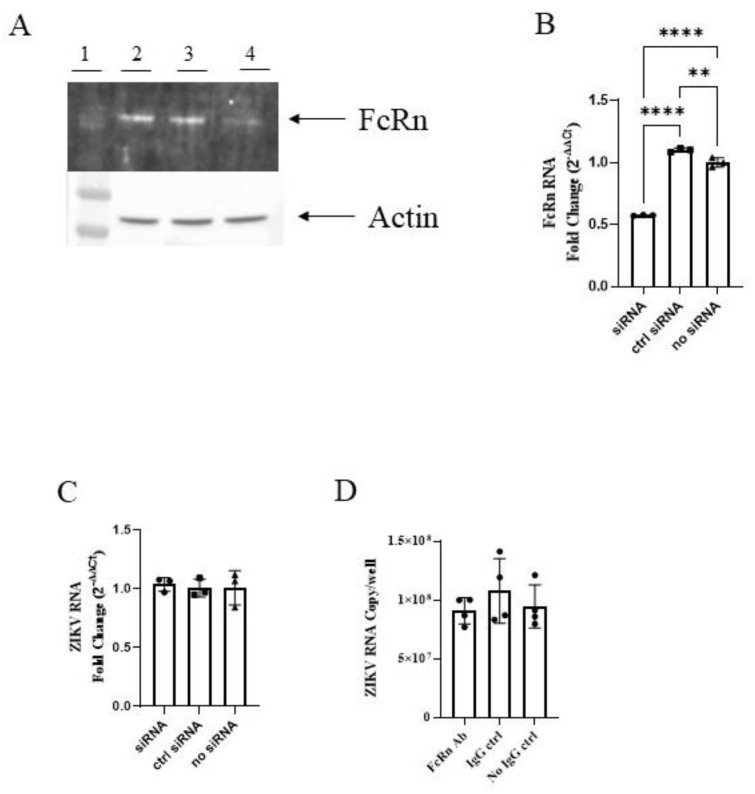
siRNA knockdown of FcRn in BeWo cells does not reduce ZIKV infection. (**A**) Western blot demonstrating knock-down of FcRn receptor expression in BeWo cells. Lane 1—molecular weight marker; 2—no siRNA; 3—control siRNA; 4—FcRn siRNA (**B**) RT qPCR showing knock-down expression of FcRn at the mRNA level. (**C**) No difference in ZIKV mRNA is seen in cells with siRNA knock down expression of FcRn. One representative of two independent experiments is shown (**A**–**C**), with at three biological repeats each cell culture analyzed by PCR (**B**,**C**). (**D**) No difference in ZIKV mRNA is seen in cells treated with anti-FcRn antibody before infection. One experiment, with four biological repeats was performed. The data was analyzed with ordinary one-way ANOVA, ** *p* < 0.05, **** *p* < 0.0001.

We performed similar knockdown experiments with BeWo cells of placental origin, a widely used model of the human syncytiotrophoblast. Although we were able to achieve a significant reduction in FcRn expression as visualized by Western blot (Figure 3A) and quantified by PCR (Figure 3B), no corresponding reductions in cellular ZIKV RNA were seen (Figure 3C). Similar results were seen following the pretreatment of the cells with anti-FcRn antibodies. Thus, unlike MDCK/FcRn cells, BeWo cells with reduced FcRn activity did not exhibit lower ZIKV infection. We note that FcRn abundance is larger in MDCK/FcRn cells compared to BeWo cells, reflected in the lower C_t_ values in qRT PCR for FcRn. For example, respective ΔC_t_ between FcRn and GAPDH were 4.9 and 8.7 for the MDCK/FcRn and BeWo cells, indicating ~14 times higher expression in the former than latter when normalized to the endogenous controls. In addition, BeWo cells are more permissive to infection compared to MDCK/FcRn cells as indicated by the ZIKV RNA copies in infectivity experiments (Figure 2D and Figure 3D for MDCK/FcRn and BeWo cells, respectively). Thus, the increase in infectivity due to FcRn receptor is likely a minor contributor to total infection levels in BeWo cells, and the change resulting from FcRn blockade is small and difficult to detect.

### 3.2. ZIKV Infection Reduces FcRn Expression in Placental Trophoblast BeWo Cells

**Figure 4:**
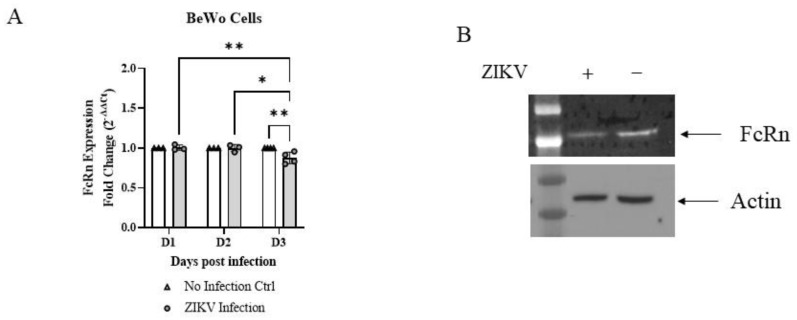
ZIKV infection reduces FcRn expression in BeWo cells. (**A**) Less FcRn mRNA is seen in the three days after ZIKV infection in BeWo cells. The averages from 3-4 independent experiments, with at least three biological repeats for experiment, are shown. (**B**) Less FcRn protein expression is seen three days after ZIKV infection in BeWo cells (three independent experiments, typical image shown). Data (**A**) was analyzed with two-way ANOVA with Tukey’s multiple comparisons test, * *p* < 0.05, ** *p* < 0.01. Signal intensities (**B**) for each band were calculated using densitometry (ImageJ) and normalized to respective loading control to allow for comparisons.

ZIKV infection in BeWo cells was associated with a small (12%), but statistically significant, decrease in FcRn mRNA levels three days post-infection (Figure 4A). The experiment was repeated four independent times, with 3–6 biological repeats in each experiment, and the results were consistent within all repeats, each showing an average decrease in the range of 4–20% (Figure 4A, average data from all experiments). FcRn protein levels also decreased, as shown by Western blot (Figure 4B), in a typical image of the three independent experiments performed. Band densities were analyzed with ImageJ to confirm the difference and resulted in a relative signal (normalized for actin) in infected and un-infected cells equal to 85% and 149%, respectively. 

The same experiment in MDCK/FcRn cells did not give conclusive results with a trend of lower FcRn mRNA (Figure S1A) but no change in the level of protein expression (Figure S1B), with relative band intensities 87% and 97% in presence and absence of infection, respectively. Infection-specific host responses in different cell lines could underlie such differences and need to be further investigated.

### 3.3. Non-Monotonous ZIKV Neutralizing Activity of Anti-Viral Antibodies

**Figure 5:**
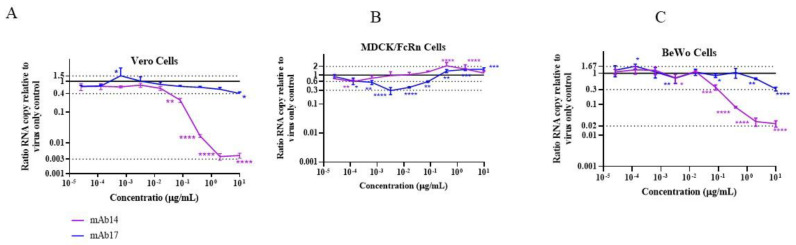
Neutralization of ZIKV infection by anti-ZIKV monoclonal antibody mAb14 (magenta) and anti-flavivirus antibody mAb17 (blue) in (**A**) Vero, (**B**) MDCK/FcRn and (**C**) BeWo cell lines. Neutralization experiments in three cell lines were repeated at least two times, representative graphs are shown. Each data point represents the average of quadruplicates. Data was analyzed with two-way ANOVA test, comparing infection at each antibody concentration to the no-antibody controls; * *p* < 0.05, ** *p* < 0.01, *** *p* < 0.0005, **** *p* < 0.0001.

We performed ZIKV neutralization assays in Vero, MDCK/FcRn and BeWo cells with two monoclonal antibodies we have studied and characterized before [12]. Both mAb14 and mAb17 antibodies exhibited neutralizing activity in Vero cells (Figure 5A, magenta and blue, respectively). As expected, anti-ZIKV mAb14 was highly neutralizing, resulting in more than a 99% reduction in the ZIKV infection at the highest concentration tested. Anti-flavivirus mAb17 antibody displayed only partial neutralization potential with a 60% decrease in infection at the highest concentration tested. In addition, one of the lowest concentrations of mAb17, 0.6 ng/mL, was associated with antibody-dependent enhancement (ADE) of infection (Figure 5A, blue graph). No ADE at any mAb14 concentrations were seen in Vero cells.

In MDCK/FcRn cells (Figure 5B), not all the antibody concentrations correlated with decreased infection. Rather, the neutralization curves were non-monotonous, with the neutralization activity first increasing and then decreasing with increased antibody levels. While lower concentrations (0.1 × 10^−3^ µg/mL for mAb14 and 0.1–3.2 × 10^−3^ µg/mL for mAb17) inhibited ZIKV infection by up to 40% and 70%, respectively, there were further elevations in the amount of mAbs-attenuated neutralization activity, resulting vfre5442ww in ADE of up to 2-fold at 0.4–10 µg/mL. The ADE of the highly neutralizing antibody mAb14 decreased at the highest concentrations tested, whereas it reached a plateau for mAb17. This non-monotonous concentration dependence in virus neutralization and ADE activity for these antibodies recapitulates the features of the bimodal Zika viral entry in the presence of these antibodies in this cell line, as we previously reported [12]. 

The neutralization behavior in BeWo cells had characteristics of both Vero and MDCK/FcRn cell lines (Figure 5C). As in Vero cells, we observed transient ADE at one of the lowest mAb17 concentrations (0.1 × 10^−3^ µg/mL) and no ADE for mAb14. Additionally, at the highest concentration tested (10 µg/mL) for mAb14 and mAb17, respective reductions of, 98% and 70% were seen in viral RNA compared to the no-antibody controls, with largely monotonous neutralization curves at concentrations higher than 80 × 10^−3^ µg/mL. On the other hand, like in MDCK/FcRn cells, at the intermediate concentration of 16 × 10^−3^ µg/mL, antibodies had no effect on viral RNA levels, whereas the antibody dilutions bracketing this value, 80 and 3 × 10^−3^ µg/mL, were neutralizing. This was especially evident for mAb14 where 80 and 3 × 10^−3^ µg/mL correlated with 70% and 30% reductions in ZIKV RNA levels, respectively—a statistically significant finding. However, unlike MDCK/FcRn cells, an increase in antibody concentrations above 80 × 10^−3^ µg/mL resulted in a decrease in infection, with neutralization curves resembling those seen in Vero cells.

### 3.4. Discussion

FcRn receptors have multiple functions in health and disease. Under normal physiologic conditions, they prolong the half-life of the IgG and albumin via pH-dependent rescue from catabolism. This can play a role in antigen presentation and the clearance of immune complexes [14,15]. Alone or in concert with other receptors, it mediates placental transfer of IgG antibodies during pregnancy [16,17]. To better understand and characterize these processes, we and others have used FcRn-expressing and -overexpressing cells [18,19,20] as a model system. Using one such model, we found that ZIKV replication was higher in MDCK/FcRn compared to the MDCK/vector stable cell lines (Figure 1). To ascertain that this observation was not a cell selection-related artifact, we performed siRNA knockdowns and antibody blockades of FcRn. As before, lower expression of FcRn resulted in reduced ZIKV RNA production, clearly implicating FcRn in ZIKV infectivity (Figure 2C). This finding is in alignment with our previous observation, that both ZIKV and its envelope glycoprotein E can enter and passage through MDCK/FcRn cells at higher levels than MDCK/vector cells [12]. It is possible that E protein interacts directly with FcRn, as has been reported for other viruses [21,22]; however, our binding studies to detect direct interaction of purified envelope glycoprotein and FcRn did not yield positive results (data not shown).

We also found that, in placental trophoblast BeWo, but not in MDCK/FcRn cells, ZIKV infection itself downregulated the expression of FcRn at the mRNA (Figure 4A) and protein level (Figure 4B), further underscoring the interplay between the infection and FcRn expression. We are not the first to observe a lower expression of FcRn in cell lines after infection by a virus. Cytomegalovirus (CMV) infection downregulates FcRn expression in multiple cell types at the protein level [20] via its ubiquitination and proteolysis in the ER. Other pathways that have been shown to be dysregulated secondarily to ZIKV infection, and could possibly play a role here, are gene methylation patterns [23] and changes in microRNA (miRNA) networks [24]. Either or both processes could be involved in the changes in receptor expression we observed. It is also notable that both methylation and miRNA signatures can exhibit different phenotypes in various susceptible cell types, which can explain the differences we saw in the two cell lines tested. To this point, a recent study in mice found increased FcRn mRNA in whole placentas three days post-ZIKV infection. Under further analysis, the elevated expression was only seen in fetal endothelial cells, but not in the syncytiotrophoblast, where a trend towards lower FcRn was observed [11].

More studies are needed to investigate if the ZIKV-mediated FcRn downregulation we observed in syncytiotrophoblast BeWo cells in vitro, can occur in vivo, especially in infected trophoblasts, placenta immune cells and maternal endothelia. If this is the case, a reduction in the FcRn expression can have clear implications during ZIKV infection. Systemically and locally, it would mediate a more rapid degradation of antibodies, including those used for anti-viral therapy. On the other hand, an even lower concentration of antibodies will exist where they are needed most, in the fetal side of the maternal-fetal unit, making it more susceptible to infection. It has not escaped our attention that both CMV and ZIKV, two viruses reported to downregulate FcRn levels in placenta, are teratogens, with tropism for both the placenta and developing fetus.

Finally, we found that two anti-viral antibodies of different specificity neutralize Zika infection in cells overexpressing FcRn (Figure 5B), and in some extent placenta cells (Figure 5C), in a non-monotonous manner. Specifically, increasing antibody concentration resulted in lower neutralization and, in MDCK/FcRn cells, in enhancement of infection. Such ADE has been shown to occur for antibodies targeting various viruses, including flaviviruses [25] and coronaviruses [26], and is mediated by antibody binding to Fc-γ receptors. Our previous [12] and current studies provide data to show that infection enhancement can also occur in cells that express FcRn. In fact, neutralization curves we observed in MDCK/FcRn cells were strikingly similar to those seen with MERS-CoV virus in cells co-expressing Fc-γRIIa and DPP4, the MERS-CoV receptor [26], indicating similar mechanisms are at play. Differences in binding affinities of antibodies to envelop glycoprotein E and FcRn likely explain the concentration dependence we observed. Both antibodies we used can bind to E protein at affinities that are 0.6–2.5 nM [12], whereas the FcRn–Fc affinity is at least one or two orders of magnitude lower, depending on pH [27]. Thus, lower antibody concentrations would favor their binding to envelope glycoprotein, the blockade of viral entry and reduction in infectivity. At higher antibody levels, FcRn-Fc binding would become important allowing antibody–virus complexes to enter cells, resulting in lower neutralization. Occurring simultaneously, each of them driving the infection in opposite directions, these processes would give rise to the non-monotonous neutralization curves we observed. At the highest concentrations, a saturation of FcRn will occur resulting in a plateau and the eventual decrease in ADE in MDCK/FcRn cells. This also explains the smaller abrogation of neutralization we saw in BeWo placenta cells, which express FcRn, but not at the artificially high levels of engineered cells. We also note that other, Fc receptor-independent mechanisms for ADE are possible [28,29]. Mechanisms such as these could underlie the transient enhancement of infection observed at sub-nanogram levels of the cross-reactive anti-flavivirus antibody mAb17, but not with anti-ZIKV specific antibody.

## 4. Conclusions

Our studies highlight the underappreciated role that FcRn plays in ZIKV infection. On one hand, as we show in engineered cells with high expression levels of FcRn, it is associated with higher susceptibility to infection. On the other hand, FcRn expression can be downregulated by ZIKV infection. The addition of antibodies that neutralize ZIKV in cells that do not express FcRn can cause the enhancement of infection in non-immune cells that overexpress this receptor. Given the abundance of FcRn in placenta, these findings draw attention to mechanisms that could enhance ZIKV ability to both infect and pass the placenta. Thus, it is critical to understand and assess the role of antibodies intended for anti-ZIKV prophylaxis and therapy in pregnant women prior to performing clinical trials.

## Figures and Tables

**Table 1:** Primers for qRT PCR.

ZIKV 1086	CCGCTGCCCAACACAAG
ZIKV 1162c	CCACTAACGTTCTTTTGCAGACAT
ZIKV 1107 (Probe)	AGCCTACCTTGACAAGCAGTCAGACACTCAA
Canine GAPDH Forward	GCAAAGTGGATATTGTCGCC
Canine GAPDH Reverse	TTTCCCGTTCTCAGCCTTG
Canine GAPDH Probe	TGCCGTGGGTAGAATCATACTGGAAC
Human GAPDH Forward	CCACTCCTCCACCTTTGAC
Human GAPDH Reverse	ACCCTGTTGCT GTAGCCA
Human GAPDH Probe	TTGCCCTCAACGACCACTTTGTC
Human FcRn Forward	CTCTGTTGTGGAGAAGGATGAG
Human FcRn Reverse	CGGTGGCTGGAATCACATTTA
Human FcRn Probe	TTGGATCTCCCTTCGTGGAGACGA

## Data Availability

The datasets used and/or analyzed during the current study are available from the corresponding author on reasonable request.

## Supplementary Materials

The following are available online at https://www.mdpi.com/article/10.3390/vaccines10122059/s1, Figure S1. ZIKV infection does not change FcRn expression in MDCK/FcRn cells. A: No significant change in FcRn mRNA is seen after ZIKV infection in MDCK/FcRn cells (four independent experiments, at least two biological repeats for experiment). B: No change in FcRn expression is seen in MDCK/FcRn cells three days after the infection, two independent experiments.
