# Supplementary material for: Zika Virus Infection and Antibody Neutralization in FcRn Expressing Placenta and Engineered Cell Lines
Source: Vaccines (Basel). 2022 Nov 30;10(12):2059. doi: 10.3390/vaccines10122059 (PMC9781090; doi:10.3390/vaccines10122059)
Supplement: Supplementary file 1 [file vaccines-10-02059-s001.zip › vaccines-2027190-supplementary.pdf]

Supplementary Materials:

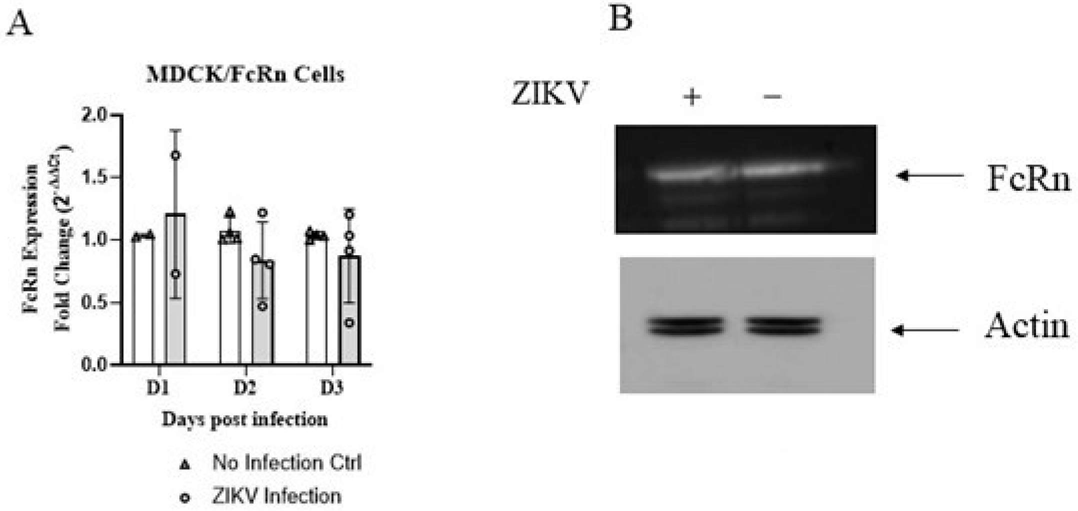

Figure S1. ZIKV infection does not change FcRn expression in MDCK/FcRn cells. A: No significant change in FcRn mRNA is seen after ZIKV infection in MDCK/FcRn cells (four independent experiments, at least two biological repeats for experiment). B: No change in FcRn expression is seen in MDCK/FcRn cells three days after the infection, two independent experiments.
